# Stevens–Johnson syndrome–toxic epidermal necrolysis (SJS–TEN) overlap associated with carbamazepine use

**DOI:** 10.4103/0019-5545.55961

**Published:** 2005

**Authors:** P.N. Suresh Kumar, Biju Thomas, Kishore Kumar, Shibu Kumar

**Affiliations:** * Director, Institute of Mental Health and Neurosciences, Kozhikode; ** Consultant Psychiatrist, Institute of Mental Health and Neurosciences, Kozhikode; *** Medical Officer, Institute of Mental Health and Neurosciences, Kozhikode; **** Medical Officer, Institute of Mental Health and Neurosciences, Kozhikode

**Keywords:** Stevens-Johnson syndrome–toxic epidermal necrolysis overlap, carbamazepine

## Abstract

A benign pruritic rash occurs in 10%–15% of persons treated with carbamazepine. A small fraction of them may experience life-threatening dermatological syndromes such as exfoliative dermatitis, erythema multiforme, Stevens–Johnson syndrome (SJS) and toxic epidermal necrolysis (TEN). The case of an 18-year-old female suffering from bipolar affective disorder (mania) who was being treated with carbamazepine, lithium, chlorpromazine and benzhexol is presented. After 10 days of treatment, she developed high-grade fever and mucocutaneous manifestations of SJS–TEN overlap. She was treated in hospital with systemic corticosteroids, antibiotics, intravenous fluids and other supportive measures, and recovered after 3 weeks.

## INTRODUCTION

Carbamazepine is an iminodibenzyl drug initially introduced as an anticonvulsant. Its beneficial effects on mood were noted in the early 1960s and currently it is used as a first-line agent along with lithium and valproic acid for the treatment of bipolar disorder.^1^ A benign pruritic rash occurs in 10%–15% of persons treated with carbamazepine. Approximately 3 persons per million per week may experience life-threatening dermatological syndromes such as exfoliative dermatitis, erythema multiforme, Stevens–Johnson syndrome (SJS) and toxic epidermal necrolysis (TEN) with the use of carbamazepine.^2^ Based on the pathogenesis, erythema multiforme, SJS and TEN are classified as non-immunological cutaneous drug reactions due to the deficiency of an enzyme or protein causing a specific, genetically determined defect in the individual's ability to detoxify toxic reactive drug metabolites.^3^

Erythema multiforme is an acute, self-limiting inflammatory disorder of the skin and mucous membranes with distinct target/iris lesions, usually acrally distributed, and accompanied by sore throat and malaise. SJS is a blistering disorder, more severe than erythema multiforme. It is characterized by mucosal erosions at two or more sites, small blisters or purpuric macules or atypical target lesions on the skin with rare areas of confluence followed by detachment of 10% or less of body surface area. Fever and lesions of the respiratory and gastrointestinal tract are seen in 10%–30% of patients. TEN is the most serious cutaneous drug reaction, and may be fatal. It is characterized by the acute onset of skin and mucosal lesions as in SJS, but with confluent erythema, large sheets of necrotic epidermis and total detachment of the skin over more than 30% of the body surface area. Fever, lymphadenopathy, hepatitis, nephritis, carditis, eosinophilia and atypical lymphocytes are present in 30%–50% of patients.

An overlap of SJS–TEN has the characteristics of both SJS and TEN with the involvement of 10%–30% of the body surface area, epidermal detachment, fever and malaise.^4^ The disease may occur as a primary skin disorder, or as a skin manifestation of systemic infections, malignant or chronic disease of the internal organs, or as a reaction to various drugs.^5^

Rzany *et al*. in a case–control study reported the risk of developing SJS and TEN for various antiepileptic agents. The univariate relative risk (RR) for 8 weeks or less of use was 120 (CI: 34–infinity) and the univariate RR for more than 8 weeks of use was 7.0 (CI: 2.4–21.0) for carbamazepine.^6^ Roujeau and Stern^7^ conducted a case-control study to quantify the risks associated with the use of specific drugs. Drug use before the onset of disease was compared in 245 people who were hospitalized because of TEN or SJS and 1147 patients hospitalized for other reasons. The crude RR for SJS or TEN was as follows—carbamazepine: 90 (95% CI: 19–infinity); phenytoin: 53 (95% CI: 11–infinity); phenobarbital: 45 (95% CI: 19–108) and valproic acid: 25 (95% CI: 4.3–infinty).^7^ No risk statistic is available regarding the risk of SJS–TEN overlap associated with anticonvulsants.

We report the case of a psychiatric patient who developed SJS–TEN overlap due to carbamazepine use.

## THE CASE

An 18-year-old married woman presented to a private psychiatry hospital in Kozhikode with a history of reduced sleep, increased talk, irritability, and aggressive and abusive behaviour of 2 weeks' duration. She was treated with lithium (800 mg/day), carbamazepine (600 mg/day), chlorpromazine (200 mg/day) and benzhexol (4 mg/day). She had a history of a manic episode that occurred 2 years back but was not on any medicines for the past 1 year. This was the second episode. Ten days after starting the medication, she developed high-grade, intermittent fever and erythematous maculopapular lesions over the neck and face spreading to other parts of the body. By day 13, the lesions had spread all over the body including the palms and soles, involving more than 90% of the total body surface area. The distribution of the lesions was symmetrical. A mucopurulent discharge was seen on the oral, nasal and conjunctival mucous membranes and there was haemorrhagic crusting of the lips. The eyelids were matted with a purulent discharge. The Nikolsky sign was found to be positive. By day 14, many lesions had become bullae, and bullous lesions involved about 20% of the body surface area.

She was admitted to the Dermatology ward of the Calicut Medical College on the same day and was diagnosed as SJS–TEN overlap. All psychiatric medications were discontinued at the time of admission. All relevant investigations were within normal limits. She was treated with parenteral and oral steroids, antibiotics, intravenous fluids and other supportive measures. The dose of steroids required frequent adjustments and they were discontinued within 3 weeks. As her psychiatric symptoms increased and became unmanageable, she was transferred to the Psychiatry ward on day 8 of admission and treated with a daily dose of chlorpromazine (300 mg), haloperidol (15 mg), benzhexol (6 mg) and clonazepam (3 mg). Her mucosal and skin lesions began subsiding after 3 weeks of inpatient treatment and there were no sequelae.

## DISCUSSION

SJS and TEN are closely related severe, episodic, acute, mucocutaneous intolerance reactions, most often elicited by drugs and less often by infections. The term SJS is reserved for cases with <10% detachment of the body surface and TEN for those with >30%. Those with detachment of 10%–30% are labelled as SJS–TEN overlap. Although somewhat artificial, this classification is useful for epidemiological purposes because the percentage of body surface area involved is a major prognostic factor in SJS–TEN overlap.^8^

Roujeau and Stern^7^ described the following algorithm for implicating a drug as the cause of an adverse drug reaction. (i) Alternative causes, especially infections, should be excluded; (ii) the interval between the introduction of a drug and the onset of a reaction should be examined (for drug-induced SJS, it is 1–3 weeks); (iii) any improvement after drug withdrawal should be noted; (iv) the physician should determine whether similar reactions have been associated with the same compound; and (v) any reaction on re-administration of the drug should be noted.

Our patient had all the features^7^ required to implicate carbamazepine as the cause of the SJS–TEN overlap. Alternative causes such as infections were ruled out, the time of onset of the adverse reaction was 10 days after starting the medication (1–3 weeks for SJS), the patient improved after withdrawal of carbamazepine, and carbamazepine is already known to cause SJS–TEN overlap. Regarding the last criterion of reaction to re-administration of the offending drug, this was not resorted to because there are reports of fatalities on re-exposure, which does not justify its use for diagnostic purposes.^9^ Reinstitution of the other drugs she had been taking after the skin lesions subsided did not produce any adverse reactions and, by exclusion, this shows that carbamazapine must have been the causative agent for producing the SJS–TEN overlap.

SJS–TEN overlap is treated by first stopping all suspected medications followed by administration of steroids, broad-spectrum antibiotics and supportive measures. The role of steroids in the treatment of SJS–TEN overlap is controversial; some call it ‘detrimental’^7^ and others ‘life-saving’.^10^ However, the prevailing consensus seems to be that systemic glucocorticoids are justified in the early stages of SJS–TEN overlap. They should be given in doses of 80–120 mg of methylprednisolone orally for several days until disease progression has ceased and then tapered quickly but cautiously.^8^ In the case of our patient, she was started on systemic glucocorticoids, which gave excellent results.

Though there are case reports of SJS occurring in schizophrenia,^11^ bipolar affective disorder^12^ and during a manic episode^13^ treated with carbamazapine, we were unable to find reports on SJS–TEN overlap due to carbamazepine in the literature from India and abroad. This suggests that SJS–TEN overlap is an underrecognized and underreported condition.

While carbamazepine is increasingly gaining acceptance as a first-line mood stabilizer in addition to being an adjuvant in other psychiatric conditions, one has to stress that the awareness of its potentially fatal side-effects, such as SJS–TEN overlap, would go a long way in its judicious use for the benefit of patients.
